# Correction to “Genetic evolution and relapse‐associated mutations in adult T‐cell acute lymphoblastic leukemia patients treated in PETHEMA trials”

**DOI:** 10.1002/hem3.70333

**Published:** 2026-03-10

González‐Gil C, Lopes T, Morgades M, et al. Genetic evolution and relapse‐associated mutations in adult T‐cell acute lymphoblastic leukemia patients treated in PETHEMA trials. *HemaSphere*. 2025;9(5):e70148. doi:10.1002/hem3.70148.

In the author listing of the manuscript, the name of one author was not included in the original publication. As such, Caterina Mata is now included as a co‐author.

Additionally, in the Author Contributions section, the name Manuel Fernández‐Delgado was mistakenly listed as Carolina Cañigral and has been corrected. Also, the contributions and disclosures of Caterina Mata have been updated.

The original article has been updated. We apologize for this error.

